# Modelling of autogenerative high-pressure anaerobic digestion in a batch reactor for the production of pressurised biogas

**DOI:** 10.1186/s13068-022-02117-x

**Published:** 2022-02-18

**Authors:** Carmen De Crescenzo, Antonia Marzocchella, Despina Karatza, Antonio Molino, Pamela Ceron-Chafla, Ralph E. F. Lindeboom, Jules B. van Lier, Simeone Chianese, Dino Musmarra

**Affiliations:** 1 Department of Engineering, University of Campania Luigi Vanvitelli, Via Roma 29, 81031 Aversa, Italy; 2 Department of Sustainability, ENEA, Italian National Agency for New Technologies, Energy and Sustainable Economic Development, CR Portici Piazzale Enrico Fermi, 1, 80055 Portici, NA Italy; 3 Sanitary Engineering Section, Faculty of Civil Engineering and Geosciences, Department of Water Management, Delft University of Technology, Stevinweg 1, 2628 CN Delft, Netherlands

**Keywords:** Autogenerative high-pressure anaerobic digestion (AHPD), Pressurised biogas, ADM1-based kinetic model, Kinetic and biological parameters assessment, Sensitivity analysis, Batch operation

## Abstract

**Background:**

Pressurised anaerobic digestion allows the production of biogas with a high content of methane and, at the same time, avoid the energy costs for the biogas upgrading and injection into the distribution grid. The technology carries potential, but the research faces practical constraints by a.o. the capital investment needed in high-pressure reactors and sensors and associated sampling limitations. In this work, the kinetic model of an autogenerative high-pressure anaerobic digestion of acetate, as the representative compound of the aceticlastic methanogenesis route, in batch configuration, is proposed to predict the dynamic performance of pressurised digesters and support future experimental work. The modelling of autogenerative high-pressure anaerobic digestion in batch configuration, which is not extensively studied and simulated in the present literature, was developed, calibrated, and validated by using experimental results available from the literature.

**Results:**

Under high-pressure conditions, the assessment of the Monod maximum specific uptake rate, the half-saturation constant and the first-order decay rate was carried out, and the values of 5.9 kg COD kg COD^−1^ d^−1^, 0.05 kg COD m^−3^ and 0.02 d^−1^ were determined, respectively. By using the predicted values, excellent fittings of the final pressure, the CH_4_ molar fraction and the specific methanogenic yield calculation were obtained. Likewise, the variation in the gas–liquid mass transfer coefficient by several orders of magnitude showed negligible effects on the model predictive values in terms of methane molar fraction of the produced biogas, while the final pressure seemed to be slightly influenced.

**Conclusions:**

The proposed model allowed to estimate the Monod maximum specific uptake rate for acetate, the half-saturation rate for acetate and the first-order decay rate constant, which were comparable with literature values reported for well-studied methanogens under anaerobic digestion at atmospheric pressure. The methane molar fraction and the final pressure predicted by the model showed different responses towards the variation of the gas–liquid mass transfer coefficient since the former seemed not to be affected by the variation of the gas–liquid mass transfer coefficient; in contrast, the final pressure seemed to be slightly influenced. The proposed approach may also allow to potentially identify the methanogens species able to be predominant at high pressure.

## Background

In the last years, particular attention has been paid to the growth of renewable energy and the improvement of technologies for its production by the European Union (EU). According to the EU, 27% of all energy production should be covered by renewable energy sources within 2030. Moreover, the targets of an annual increase of 1% in renewable energy in the heating sector and 14% in renewables sources in the transport sector should be reached within 2030. In particular, advanced biofuels and biogas should be used for the 3.5% of the energy required for the transport sector [1]. In this context, anaerobic digestion (AD) is a well-established biological process that converts biodegradable substrates into biogas in the absence of oxygen. This renewable energy source, mainly consisting of methane and carbon dioxide, can be used—in internal combustion engines—for the production of electrical and/or thermal energy [2, 3]. Moreover, by separating CO_2_ from biogas, the production of biogenic methane, i.e. high purity CH_4_ gas, can be performed [4, 5].

Unlike natural gas, biogenic methane is a renewable energy source with the same quality (CH_4_ ≥ 95%). It can be used in the same natural gas applications according to national laws, i.e. it can be injected into the natural gas grid, as well as used in the transport sector [6, 7]. The specifications for the injection of biogas in the natural gas grid and its use as vehicle fuel are defined by the European Committee of Standardization [8, 9]. Lombardi and Francini [2] reported that different types of economic incentives had been developed in EU countries, such as Italy, Sweden, and Germany, in order to promote biogenic methane as a fuel. Using biogenic methane as a substitute for natural gas, instead of converting biogas into electricity/heat, allows for more primary energy savings and helps to reach the minimum target of 14% renewables in the transport sector [10, 11].

The improvement of biogas upgrading techniques is strictly connected to advantageous economic perspectives, widening the environmental and social benefits of AD [12, 13]. Several approaches can be used for removing CO_2_ from biogas, such as pressure swing adsorption, scrubbing, cryogenic and membrane separations [2, 14]. Among them, membrane separation presents the lowest overall costs, with multistage configurations able to provide methane recovery and purity of 99% and 95–99%, respectively, compared to single-stage configurations [15, 16]. However, it requires high electricity consumption, in the range 0.19–0.77 kWh/Nm^3^ due to the compression step [2], with 30–40% of the costs for the production of biogenic methane related to the compression energy [17].

Recently, considerable attention has been paid to pressurised anaerobic digestion (PAD), which consists of an AD occurring at a pressure higher than the atmospheric one. Pressurised anaerobic digestion can be performed in continuous reactors [18] or batch reactors by the addition of external gas (i.e. N_2_ or CO_2_) or by accumulating biogas in the headspace of the reactor, which leads to a gradual increase in autogenerated pressure [19, 20]. Unlike the temperature change, a pressure increase does not directly impact the biological part of the process; otherwise, it influences the final composition of the biogas, leading to a higher concentration of methane in the biogas, even reaching 90% [21].

Lindeboom et al. [22] reported the potential of autogenerative high-pressure digestion (AHPD) of sodium acetate, performed in batch reactors observing CH_4_ contents higher than 90% at pressures exceeding 20 bar. While Lemmer et al. [23] observed in AHPD of maise silage and a mixture of grass and maise silage, the composition of biogas is stable at a defined working pressure (1 to 9 bar) despite the increase in organic load rate. Values of the content of CH_4_ of about 90%v/v, at an operating pressure of 50 bar, were found by Merkel et al. [24] with continuous high-pressure anaerobic digestion of maize silage and grass. Literature findings showed that pressure has a more significant impact on the biogas composition than the organic loading rate [25]. Therefore, it seems plausible that the applied operating pressure, the initial substrates, and their degree of dissociation and reduction influence the composition of the biogas produced [26].

Pressurised anaerobic digestion is an attractive and potentially cost-effective pressurised biogas production technology, with the added advantage that by increasing pressure, the solubilisation of CO_2_, as compared to CH_4_, increases; consequently, the biogas is also upgraded [19, 22, 27, 28]. Therefore, with PAD technology, it is possible to obtain biogas with a high content of CH_4_ and, at the same time, avoid the costs of energy required for the biogas upgrading and injection into the distribution grid. The pressure of biogas produced in PAD significantly reduces the energy needed for injecting the produced biogas into the gas grid by 45–60% [21].

The innovative but complex aspects of the PAD process require modelling investigations that lead to a better understanding and prediction of the behaviour of pressurised digesters, and ultimately design improvements could be proposed. Among existing AD models, the anaerobic digestion model No.1 (ADM1) by Batstone et al. [29] is the most studied and used for modelling anaerobic digestion processes. Blumensaat and Keller [30] presented a process model to simulate the dynamic behaviour of a semi-continuous pilot-scale process for anaerobic two-stage digestion of sewage sludge performed at atmospheric pressure. The implemented model, based on the IWA Anaerobic Digestion Model No.1 (ADM1), was used to support experimental investigations of the anaerobic two-stage digestion process. Fezzani and Cheikh [31] demonstrated that the modified ADM1 could adequately simulate the steady-state behaviour of anaerobic semi-continuous tubular digesters treating in co-digestion of olive mill wastewater with olive mill solid waste at thermophilic temperature and atmospheric pressure. The simulation results showed that the modified ADM1 could reasonably well predict the steady-state results of gas flows, methane and carbon dioxide contents, pH and total volatile fatty acids. Wichern et al. [32] applied ADM1 for modelling grass silage fermentation carried out in two semi-continuous digesters at the mesophilic condition and atmospheric pressure. The model was calibrated both manually and with the help of a Genetic Algorithm in MATLAB/SIMULINK.

Few works simulating the PAD processes in batch reactor systems are reported in the literature. Antonopoulou et al. [33] used the Anaerobic Digestion Model No. 1 to estimate the kinetic parameters for hydrogen and organic acids consumption through fitting of the model equations to the data obtained from batch experiments carried out on acidified sorghum extract generated from a hydrogen-producing bioreactor in a two-stage anaerobic process at atmospheric pressure, showing good agreement between experimental data and modelling results. In the paper by Souza et al. [34], the ADM1 was used as a tool to assess the effects of thermal pre-treatment and hydraulic retention times on the performance of three batch pilot-scale digesters fed with mixed sludge with/without pre-treatment applied to the waste activated sludge fraction and operated at atmospheric pressure. Model calibration was carried out by using data from biochemical methane potential tests, and the validation of the calibrated model reported a good accuracy of both average and accumulated CH_4_ production lower than 15% in all cases. Manjusha and Beevi [35] proposed a modified version of the ADM1 to model and simulate anaerobic digestion of batch study at atmospheric pressure and found out how the factors such as pH and volatile fatty acid affect the daily biogas production. A kinetic model based on ADM1 was proposed by Huang et al. [36] to describe the acetate-type fermentation in an anaerobic membrane bioreactor under acidic pH conditions. The modelling results revealed that the fermentation pathway was closely associated with the pH-dependent hydrogenotrophic methanogenesis and ethanol-producing activities. Acetate and butyrate production was thermodynamically more favourable at pH 5.0 over 4.0, resulting in CH_4_ low content. Spagni et al. [37] showed that ADM1 is suitable for modelling the biological anaerobic processes involved in a membrane-assisted bioreactor treating synthetic wastewater composed of cheese whey and sucrose. They also highlighted that, after modifications to the fractions describing the wastewater characterisation, the ADM1 was capable of reasonably fitting the experimental data with the calibration of one parameter only.

Postawa et al. [20] proposed a mathematical ADM1-based model of the AD process. They simulated two-stage autogenerative high-pressure digestion; however, as experimental results were not available, the model was utilised as a tool to provide all necessary data for the assessment of the process. Their findings recommend using the AHPD concept, as a methane content value even higher than 82% can be achieved.

Despite the extensive use of batch systems in industrial applications [38], it is worth highlighting that PAD in single batch stage reactors has not explicitly been simulated and validated in a dynamic modelling framework like the ADM1.

In this work, an AHPD model in the batch configuration is proposed to predict the dynamic performance of pressurised digesters and as a tool to maximise the mechanistic understanding of PAD for experiments that are limited by sampling constraints typically associated with PAD research. In particular, the modelling of AHPD of acetate, as the representative compound of the last step in AD, i.e. the aceticlastic methanogenesis, was developed, calibrated, and validated on experimental results by Lindeboom et al. [22]. The model was used to assess the Monod maximum specific uptake rate for acetate, the half-saturation value for acetate and the decay rate constant of microorganism species. In addition, a sensitivity analysis to determine the effects of the variation of the selected parameters, including the overall gas–liquid mass transfer coefficient, on the modelled pressure, the CH_4_ biogas molar fraction and the specific methanogenic yield (SMY) was performed. Moreover, based on the assessment of the investigated kinetic and biological parameters, the proposed approach may allow to potentially identify the aceticlastic methanogens species able to grow at high pressures.

## Results and discussion

### Model calibration and validation results

The results of different experimental studies on PAD showed a significant impact of pressure on the growth and evolution of the microbial community and the performance of the process. Several empirical studies focused on the variation in microbial composition, i.e. bacteria and archaea, dominating the process [22, 39, 40]. These studies highlighted the interaction between community composition, CH_4_ content of the biogas and rate of biogas production. Due to the effect of alkalinity of the growth medium on both the gas composition and the pH, it should also be considered to have a pronounced impact on the population dynamics [41, 42]. None of these studies focused on estimating kinetic parameters under elevated pressure, while these are highly important to predict the community composition and subsequent reactor performance [43].

**Fig. 1:**
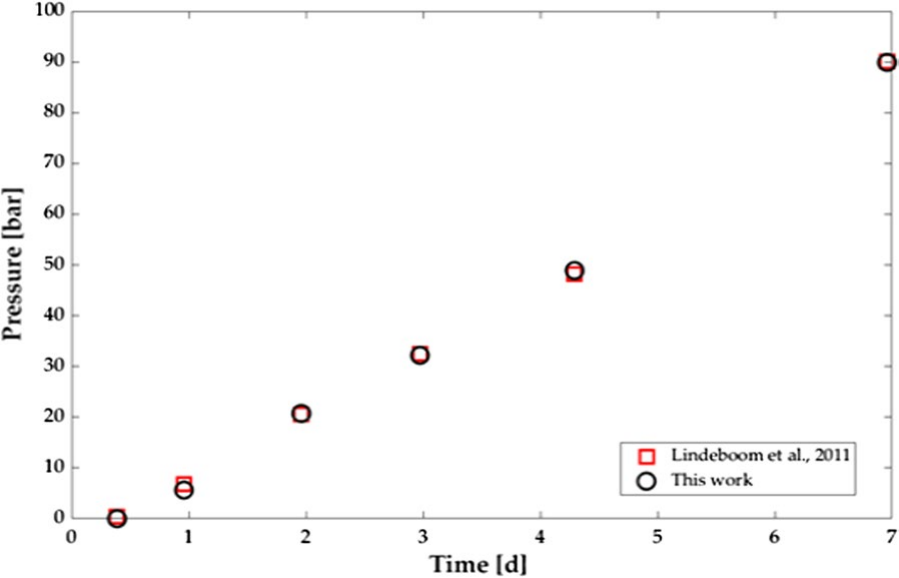
Modelling results (circle) and experimental results of Experiment no. 7 from Lindeboom et al. [22] (square) comparison for the calibration—*R*^2^ = 0.999; GA fitness function result = 0.992 bar

The comparison between the pressure monitored during Experiment No. 7 performed by Lindeboom et al. [22], which was used for calibrating the model (please, see “Model calibration and validation” section), and the predicted values of the pressure, as a function of operating time, is shown in Fig. 1. The experimental conditions of Experiment No. 7 are: reactor volume = 1.68 L; gas volume = 0.01 L; initial substrate concentration = 3 g sodium acetate COD/L; time of the experiment = 160 h; initial pH $\cong$ 7; final pH $\cong$ 7.

Experimental and simulated results are nearly overlapping, with the coefficient of determination, *R*^*2*^, equal to 0.999 and the genetic algorithm fitness function of 0.992. Parameters assessed through the calibration, including the comparison with literature values, are shown in Table 1.

**Table 1:** Calibration results

Parameter	Predicted AHPD value	Reported value at atmospheric pressure	Ref.
*k*_*m,ac*_ (kg COD kg COD ^−1^ d^−1^)	5.9	8	[29]
*K*_*S,ac*_ (kg COD m^−3^)	0.05	0.15	[29]
*k*_*dec,Xac*_ (d^−1^)	0.02	0.02	[29]

As shown, *k*_m,ac_, *K*_S,ac_*, k*_dec,*X*ac_ are comparable with literature values reported for atmospheric AD. Furthermore, the values seem in the same range, as the Monod parameters reported, for well-studied methanogens under atmospheric pressures [44].

The predicted value of the half-saturation is only slightly higher than the reported value for *Methanosaeta.* Therefore, we may speculate that the culture was dominated by *Methanosaeta-*like aceticlastic methanogens, which generally predominate at low substrate concentrations. Interestingly, a later AHPD reactor, inoculated with crushed anaerobic granules from a fruit juice wastewater EGSB reactor was also dominated by *Methanosaeta concilii-*like methanogens [40]. The same species was found by Aoyagi et al. [45], which identify acetate-degrading microorganisms actively in the anaerobic membrane bioreactor system treating a model slurry of high-strength organic solid waste by high-sensitivity stable isotope probing of rRNA. The same achievement was reported by Zhao et al. [46], which compared the microbial communities in anaerobic digesters treating high alkalinity synthetic wastewater at atmospheric and high-pressure (11 bar).

However, in this specific analysed experiment by Lindeboom et al. [22], no molecular characterisation of the microbiome was performed. Therefore, the predictive capacity of the model towards the predominant microorganisms remains an interesting feature that needs to be further validated in future work. In particular, it would need to be verified by an in-depth Next Generation Sequencing-based study of the reactor microbiome. It would also be interesting for future work to explore the competition between methanogens based on growth rate and/or substrate affinity in the overall pressure accumulation based on estimated kinetic parameters.

Moreover, a slight decrease of the final pH as a function of the operating time, as compared to the final pH observed during the experimental investigation, with the predicted value of about 6, was found. The same trend was reported by Atallah et al. [47], which optimised the production of methane resulting in the reduction of pH along time and pointing out that the prediction of the intermediary outputs was strongly affected by the parameter optimised. Additionally, Garcia-Gen et al. [48] correctly simulated the composition of the produced biogas, although the pH values in the reactor were significantly underestimated. However, the predicted value of the final pH (pH $\cong$ 6) is coherent with the *Methanosaeta-*like aceticlastic methanogens, although the optimal pH level was 7.6–7.7 [49, 50].

The model was validated by simulating experiments No. 4, 5, 6, and 7 using the calibrated parameters (*k*_m,ac_, *K*_S,ac_, and *k*_dec,*X*ac_). Results of the validation of the model in the simulation of AHPD are shown in Table 2. For each experiment, a run time equal to the run times reported in Table 7 was used in order to simulate the same conditions.

**Table 2:** Experimental and simulated final pressure and CH_4_ content in biogas in experiments No. 4, 5, 6 and 7 [22] reported in Table 5

Experiment No. [22]	Final pressure (bar)			Final CH_4_ molar fraction (%)		
	Experimental [22]	Modelling	Variation (%)	Experimental [22]	Modelling	Variation (%)
4	23	22.4	+ 2.6	94	93.3	+ 0.7
5	22	21.7	+ 1.4	89	93.3	− 4.8
6	58	57.9	+ 0.2	96	95.3	+ 0.7
7	90	89.5	+ 0.6	n.a	95.3	–

*n.a.*  not available

Table 2 clearly shows that both experimental and simulated results were very similar, both in terms of the final pressure attained and the CH_4_ molar fraction of the produced biogas. For the final pressure, the variation between the experimental results and the predicted values was very limited, with the highest value of 2.6% in correspondence to Experiment No. 4; for the molar fraction of CH_4_, the highest variation of 4.8% was observed for the Experiment No. 5. The effectiveness of the ADM1 implemented in MATLAB was highlighted by Satpathy et al. [51], which proposed a modified ADM1 with lactate incorporation to describe a full-scale anaerobic reactor operated at atmospheric pressure, treating food waste and cattle manure. This model resulted in successfully improving the fit against experimental data with a difference of nearly 0.8% and 0.2% for biogas yield and methane content, respectively.

Comparing methane content at high pressures, it could be found that they are resembling. In particular, Kim et al. [52] reported that the increase of CH_4_ content in the biogas at elevated pressure is usually investigated by using continuous reactors. CH_4_ content values in the range 75–78%v/v were achieved at 9 bar in an anaerobic filter system fed with diluted maize silage and grass of 20–25 g COD/L [23, 53, 54]; moreover, it achieved 80%v/v and 90%v/v at 6 and 17 bar, respectively, from the food waste of having 100 g COD/L [55]. However, Kim et al. [52] pointed out that comparisons with former performance are not suitable in AHPD, since the CH_4_ content could be varied depending on the substrate characteristics, in particular, substrate concentration. Therefore, variability of initial COD concentration represents a further parameter to investigate.

### Specific methanogenic yield calculations

The comparison between the SMY from experimental data [22] and modelling results is shown in Table 3. The predicted SMY is similar to the SMY estimated from the experimental data, with all variations below 3.5%. It should be noted that the formation of C3–C6 VFAs was experimentally confirmed at the end of Experiment No. 6. However, since the origin was postulated to be inoculum decay, these findings have been excluded from this SMY prediction.

**Table 3:** Comparison between SMY for experimental and simulated results

Experiment No.	SMY from experiments [22] (L/kg)	SMY from modelling (L/kg)	Variation (%)
4	158.4	153.1	+ 3.3
5	86.1	89.1	− 3.5
6	21.5	21.3	+ 0.9
7	n.a	32.9	–

*n.a.*  not available

### Sensitivity analysis results

**Fig. 2:**
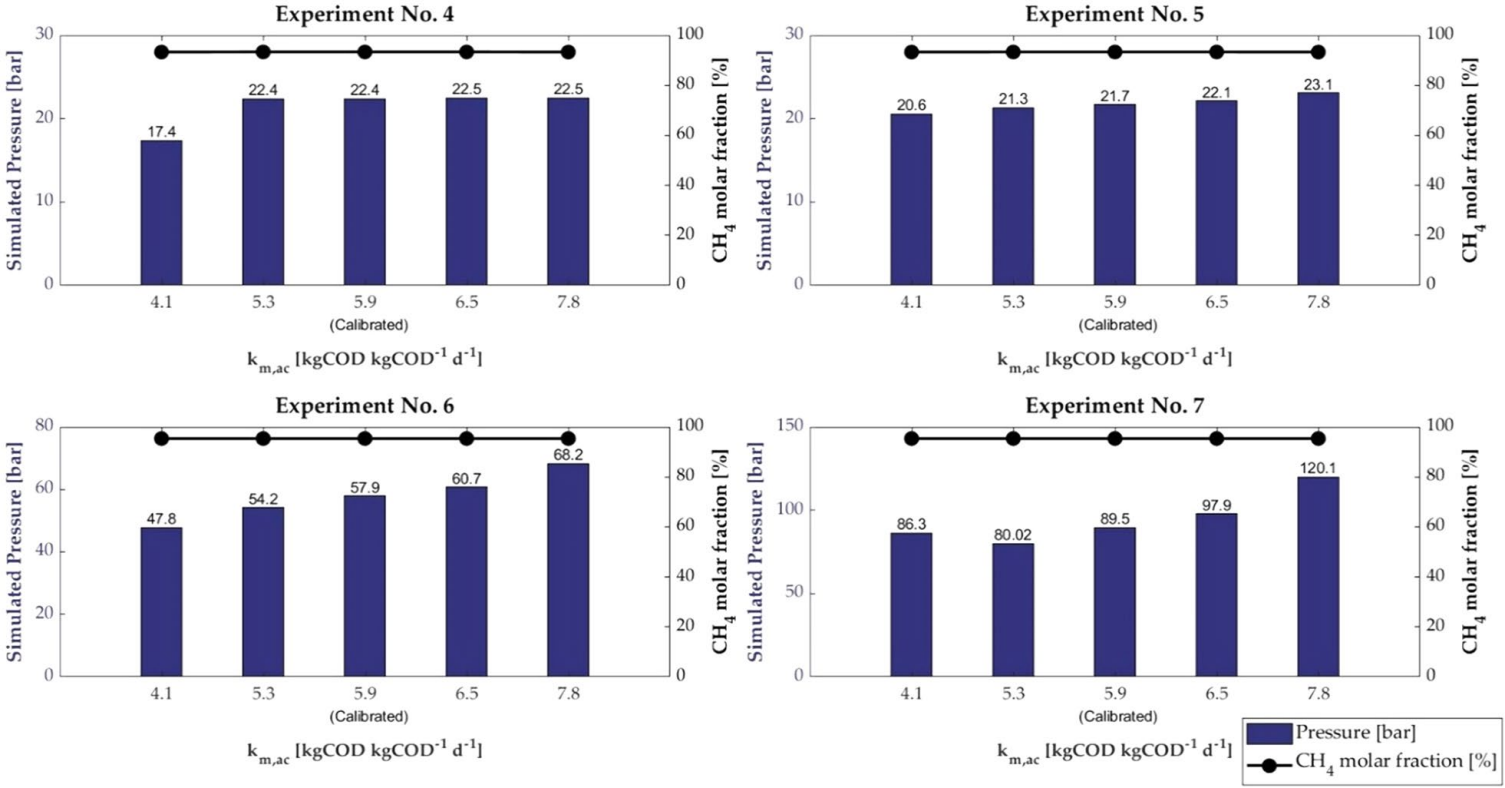
Pressure and CH_4_ molar fraction of all experiments for different value of *k*_*m,ac*_

The effect of the variation in *k*_m,ac_ in the range 4.1–7.8 kg COD kg COD^−1^ d^−1^ on the final pressure and the final CH_4_ molar fraction is presented in Fig. 2.

The highest variations in pressure, concerning the calibrated values, were observed for experiments No. 6 and 7, which correspond to the highest digestion pressure reached. This finding may be attributed to the fact that experiments No. 6 and 7 were performed at the highest initial concentration of sodium acetate, i.e. 14 g sodium acetate COD/L for both experiments, but they were performed for a different duration. In particular, Experiment No. 7, which showed the highest variation, had a total experimental run time of 170 h, while Experiment No. 6 was completed in 96 h.

The CH_4_ molar fraction was found almost the same for the different values of *k*_*m,ac*_, with an equal CH_4_ molar fraction of 93.4% for experiments No. 4 and 5, and 95.3% for experiments No. 6 and 7.

**Fig. 3:**
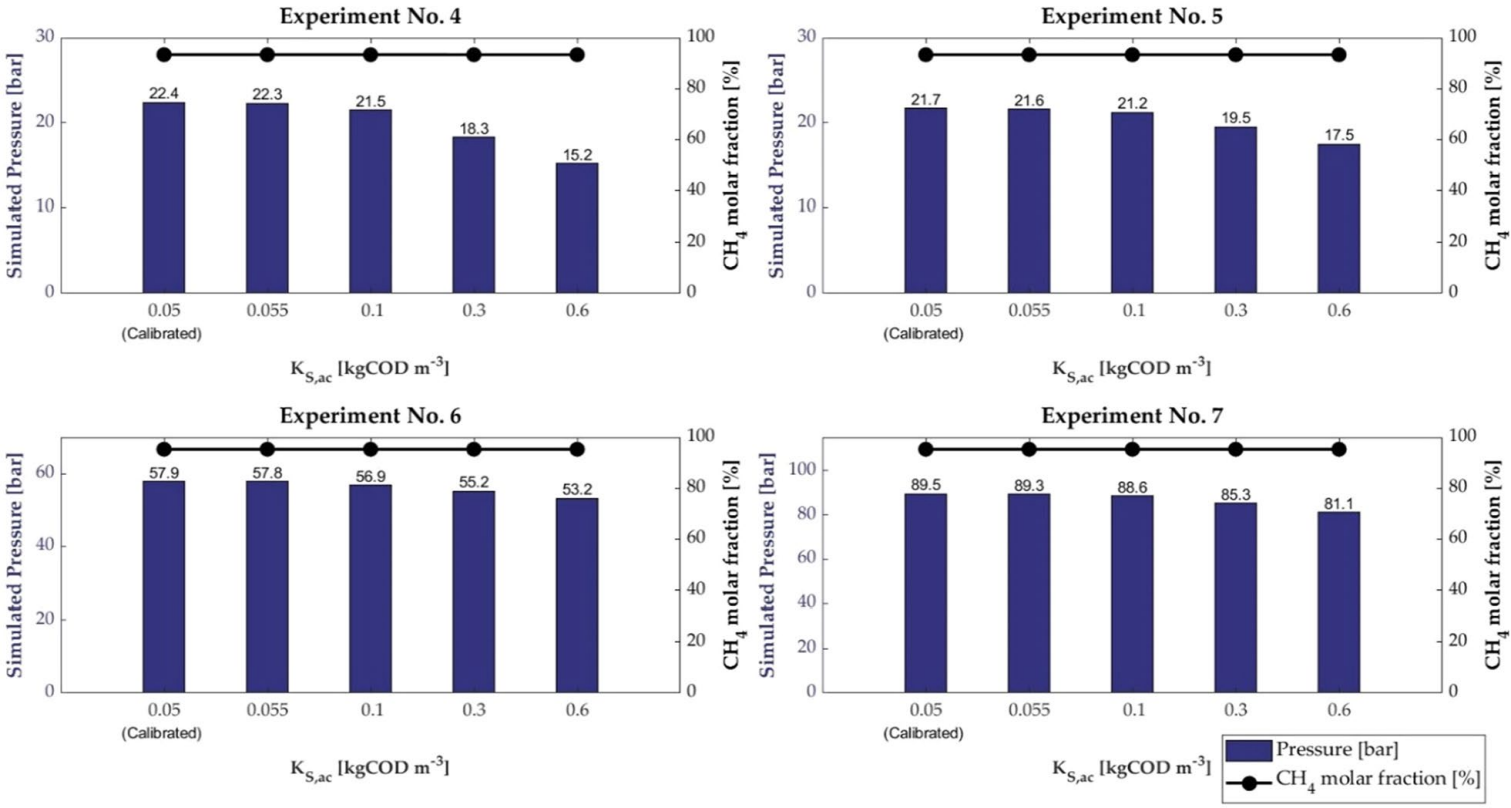
Pressure and CH_4_ molar fraction of all experiments for different value of *K*_*S,ac*_

The effect of variation in *K*_S,ac_ in the range 0.05–0.6 kg COD m^−3^ on the final pressure and the final CH_4_ molar fraction is depicted in Fig. 3.

As shown, the final pressure decreased by increasing *K*_S,ac_. The deviation from the simulated results for calibrated *K*_S,ac_ equal to 0.05 kg COD m^−3^ seems to be more relevant for Experiment No 4. This finding might be explained by the fact that Experiment No. 4 is characterised by the lowest initial concentration of sodium acetate and a relatively high run time, i.e. 160 h.

The CH_4_ molar fraction was found almost constant at a value of 93.4% for experiments No. 4 and 5 and at a value of 95.3% for experiments No. 6 and 7. Results showed that the variation in *K*_S,ac_ strongly affected the simulated pressure, as compared to the CH_4_ molar fraction, which had almost the same value as the value computed from the calibrated *K*_S,ac_ value.

**Fig. 4:**
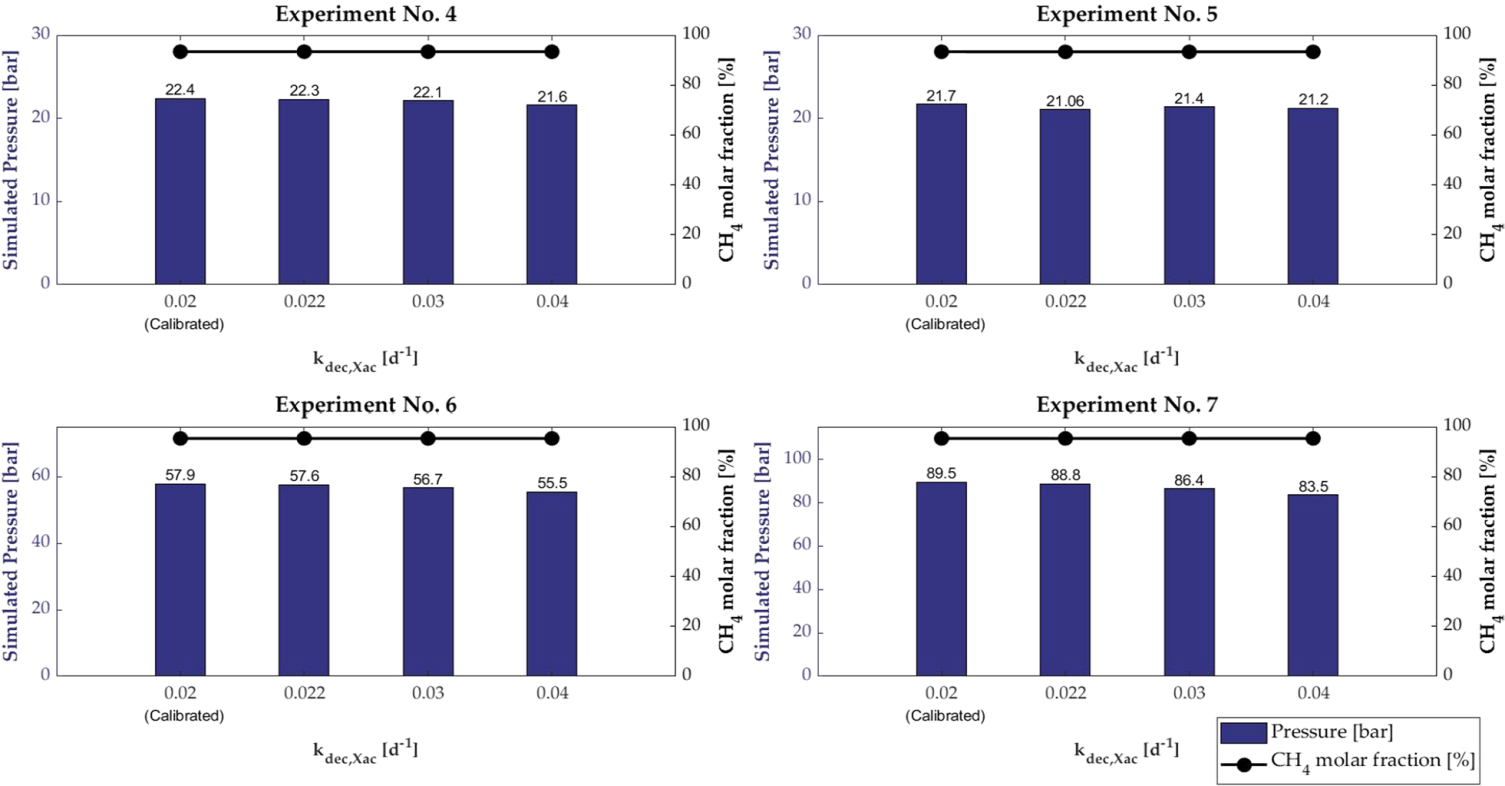
Pressure and CH_4_ molar fraction of all experiments for different value of *k*_*dec,Xac*_

The effect of the variation in *k*_dec,*X*ac_ in the range 0.02–0.04 d^−1^ on the final pressure and the final CH_4_ molar fraction is depicted in Fig. 4.

In all experiments, results showed that by increasing *k*_dec*,X*ac*,*_ the pressure decreased. The highest variation was found for Experiment No. 7, which had the most extended experimental period. The CH_4_ molar fraction showed an equal value of 93.4% for experiments No. 4 and 5 and an equal value of 95.3% for experiments No. 6 and 7.

The final CH_4_ molar fraction seemed not to be affected by the variation of the kinetic parameters highlighting that the composition of the biogas is mainly influenced by equilibrium reactions, i.e. ion association and dissociation reactions, and by gas transfer secondary. On the other hand, the final pressure was affected by the variation of the kinetic parameters investigated as: the higher the uptake rate for acetate, the higher the pressure, since the increase in the acetate uptake rate increases the methane produced, increasing in the final pressure; the higher the half-saturation value for acetate, the lower the pressure, since the increase in the acetate half-saturation value decreases the biomass growth, decreasing in the substrate consumption and the methane production; the higher the first-order decay rate, the lower the pressure, since the increase in the biomass decay rate decreases the biomass growth, decreasing in the substrate consumption and the methane production. The sensitivity analysis shows that among the investigated kinetic parameters, the Monod maximum specific substrate uptake rate significantly affects the final pressure, i.e. the production of methane in agreement with Mendes et al. [56].

The effect of the variation in liquid–gas mass transfer coefficient ${k}_{\mathrm{L}}a$ on the final pressure and the biogas CH_4_ molar fraction for Experiment No. 6 is reported in Table 4. Results show that the variation in ${k}_{\mathrm{L}}a$ had no observable effect on the CH_4_ molar fraction, highlighting that the production of biogas seems to be mainly governed by the substrate conversion kinetics, while the final pressure seemed to be slightly influenced. Nonetheless, other limitations may occur; for example, an increased CO_2_ concentration may damage the archaeal membranes with a consequent reduction in substrate uptake rate. So far, since CO_2_ has not yet been included as an inhibition parameter in the ADM1, it would be highly interesting for future work to explore the potential inhibitory role of CO_2_ [57]. Moreover, the CH_4_ molar fraction strongly depends on the type of substrate and its degree of reduction. In contrast, the influence of the microbial community will restrict itself to the observed conversion rate. At short operative run times, this could result in incomplete substrate conversion.

**Table 4:** Pressure and biogas CH_4_ molar fraction for Experiment No. 6 for different values of *k*_*L*_*a*

${{\varvec{k}}}_{{\varvec{L}}}{\varvec{a}}$ (d^-1^)	Pressure (bar)	Variation^*^ (%)	CH_4_ molar fraction (%)	Variation^*^ (%)
1	55.5	4.1	95.1	0.2
200	57.9	0.0	95.3	0.0
1000	57.9	0.0	95.3	0.0

^*^Variation calculated for the reference value *k*_*L*_*a* = 200 d^−1^

## Conclusions

The proposed simplified ADM1, with a Genetic Algorithm-based parameter estimation, adapted towards pressurised environments, can adequately predict methane production and biogas quality resulting from aceticlastic methanogenesis. It allowed to estimate the parameter values of the Monod maximum specific uptake rate for acetate, the half-saturation value for acetate and the first-order decay rate, which were comparable with literature values reported for well-studied methanogens under anaerobic digestion at atmospheric pressure. The proposed approach may also allow to potentially identify the methanogens species able to be predominant at high pressures. The value of the half-saturation constant was relatively close to literature reported values for *Methanosaeta*-like methanogens.

The sensitivity analysis showed that the final CH_4_ molar fraction seemed not to be affected by the variation of the kinetic parameters highlighting that the composition of the biogas is mainly influenced by equilibrium reactions, i.e. ion association and dissociation reactions, and by gas transfer secondary. On the other hand, the final pressure was affected by the variation of the kinetic parameters investigated.

The proposed model demonstrates that further studies using ADM1 or modified and simplified forms of ADM1 will be beneficial to increase our mechanistic understanding of the differences and similarities between conventional AD and PAD.

## Method

**Fig. 5:**
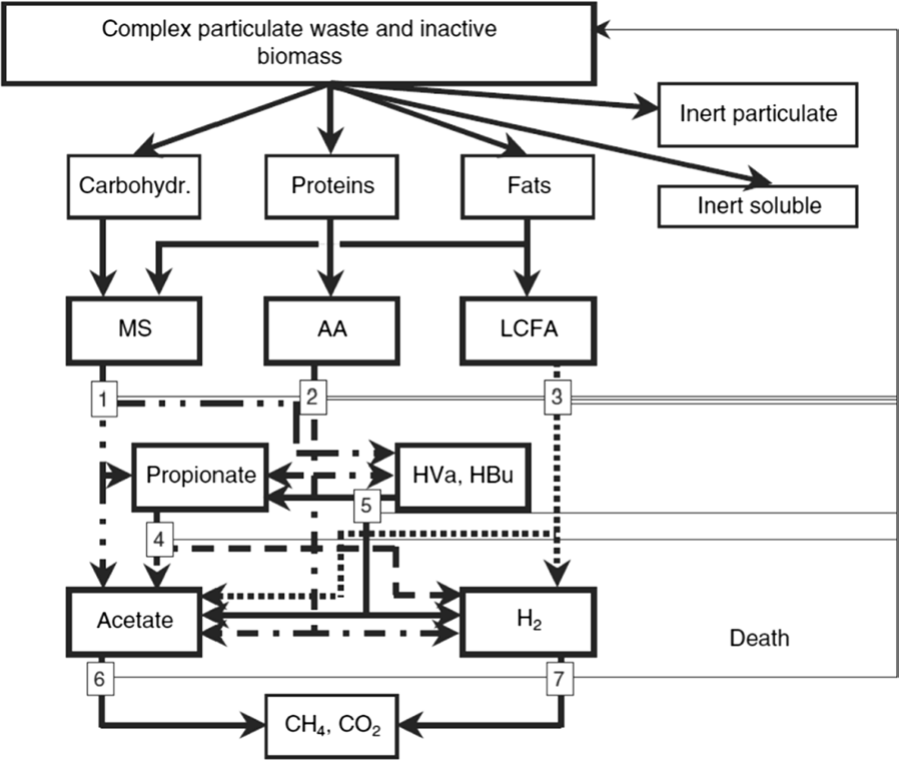
ADM1 schematisation: (1) acidogenesis from sugars, (2) acidogenesis from amino acids, (3) acetogenesis from LCFA, (4) acetogenesis from propionate, (5) acetogenesis from butyrate and valerate, (6) aceticlastic methanogenesis, and (7) hydrogenotrophic methanogenesis; MS—monosaccharides, AA—amino acids, LCFA—long-chain fatty acids. Reprinted from Water Science and Technology volume 45, issue number 10, pages 65–73, with permission from the copyright holders, IWA Publishing

The biochemical reactions that govern the AD process constitute a complex system of series–parallel reactions, which include fast liquid–liquid reactions, i.e. ion association and dissociation reactions; medium–high rates gas–liquid reactions, i.e. gas transfer and medium–low rates liquid–solid reactions, i.e. precipitation and solubilisation of ions. The original ADM1 included the first two types of reactions and was extended with a module on chemical speciation and precipitation [58]. The ADM1 simulates the degradation of complex solids into proteins, fats, carbohydrates, and inert compounds. These degradation products are then hydrolysed to amino acids (AA), long-chain fatty acids (LCFA), and monosaccharides (MS), respectively. Carbon dioxide, methane, hydrogen and water vapour are the main components of the gas phase involved in these reactions [29]. Volatile fatty acids (acetate, propionate, valerate and butyrate) and H_2_ can be generated via the acidogenic fermentation of proteins and carbohydrates [29]. Methane is produced by aceticlastic cleavage of acetate and hydrogenotrophic reduction of CO_2_ by H_2_, while the other acetotrophic route, syntrophic acetate oxidation, is not included in ADM1 [29]. Extracellular biochemical reactions are approximated by first-order rate law kinetics, while intra-cellular biochemical reactions are described by Monod-type kinetics. Substrate uptake reaction rates are proportional to the biomass growth rate and biomass concentration [59]. A complete schematisation of the main AD reaction steps is reported in Fig. 5. The ADM1 was applied to model the processes with stoichiometric coefficients, equilibrium coefficients, dynamic states and algebraic variables as proposed by Batstone et al. [29], where details about model governing equations, input parameters, and underlying assumptions are described.

Acetate is the representative substrate for the aceticlastic methanogenesis route (path 6 in Fig. 5), consisting of the anaerobic dismutation of acetate with the formation of methane and bicarbonate according to the following global irreversible reaction, which was considered in the proposed model [60]:

1 $${{\mathrm{CH}}_{3}\mathrm{COO}}^{-}+{\mathrm{H}}_{2}\mathrm{O}\to {\mathrm{CH}}_{4}+{{\mathrm{HCO}}_{3}}^{-}$$

### Autogenerative pressurised anaerobic digestion modelling: equations and parameters

The pressurised anaerobic digestion of acetate in a batch reactor under autogenerative regime was simulated by modifying the ADM1 from a continuous regime to a discontinuous one, since the original structure of the ADM1 was based on the AD of a substrate in a CSTR reactor [61], and the relevant model sections for aceticlastic methanogenesis of the ADM1 were used.

The model for acetate digestion consists of eight differential equations in six state variables and two additional variables linked to liquid–gas mass transfer: a soluble substrate (sodium acetate) ${S}_{\mathrm{ac}}$ (kg COD m^−3^), soluble methane ${S}_{{\mathrm{CH}}_{4}}$ (kg COD m^−3^), soluble inorganic carbon ${S}_{\mathrm{IC}}$ (kmol C m^−3^), particulate matter (aceticlastic methanogens) ${X}_{\mathrm{ac}}$ (kg COD m^−3^), soluble acetate ions ${S}_{\mathrm{ac}-}$ (kg COD m^−3^), soluble hydrogen carbonate ${S}_{{\mathrm{HCO}}_{3}-}$ (kg COD m^−3^), CH_4_ in the gas phase ${S}_{\mathrm{gas},{\mathrm{CH}}_{4}}$ (kg COD m^−3^), and CO_2_ in the gas phase ${S}_{\mathrm{gas},\mathrm{CO}2}$ (kmol C m^−3^). It is worth highlighting that acetic acid contribution will be marginal, i.e. about two orders of magnitude lower than acetate, given the prevailing pH.

The following equation system describes the biochemical reactions and mass transfer from liquid to gas in the liquid phase. In particular, to simulate the autogenerative high-pressure digestion in a discontinuous reactor, the ADM1 was modified by not including inlet and outlet liquid flow rates and biogas outlet flow rate:

2 $$\frac{{\mathrm{dS}}_{\mathrm{ac}}}{\mathrm{dt}}=-{\uprho }_{1},$$

3 $$\frac{{\mathrm{dS}}_{{\mathrm{CH}}_{4}}}{\mathrm{dt}}={\nu }_{1,{\mathrm{s}}_{{\mathrm{CH}}_{4}}}{\cdot \uprho }_{ 1}-{\uprho }_{ {\mathrm{T}}_{{\mathrm{CH}}_{4}}},$$

4 $$\frac{{\mathrm{dS}}_{\mathrm{IC}}}{\mathrm{dt}}={-(\nu }_{1,{\mathrm{S}}_{\mathrm{IC}}}{\cdot \uprho }_{ 1})-{\uprho }_{ {\mathrm{T}}_{{\mathrm{CO}}_{2}}},$$

5 $$\frac{{\mathrm{dX}}_{\mathrm{ac}}}{\mathrm{dt}}={\nu }_{1,X\mathrm{ac}}{\cdot \uprho }_{ 1}-{\nu }_{2,X\mathrm{ac}}{\uprho }_{ 2},$$

6 $$\frac{{\mathrm{dS}}_{\mathrm{ac}-}}{\mathrm{dt}}=-{\uprho }_{ {\mathrm{A}}_{\mathrm{ac}}},$$

7 $$\frac{{\mathrm{dS}}_{{\mathrm{HCO}}_{3}^{-}}}{\mathrm{dt}}=-{\uprho }_{ {\mathrm{A}}_{{\mathrm{HCO}}_{3}^{-}}}.$$

Two liquid–gas dynamic equations for CH_4_ and CO_2_ for a discontinuous reactor are written as in the following:

8 $$\frac{{\mathrm{dS}}_{\mathrm{gas},{\mathrm{CH}}_{4}}}{\mathrm{dt}}={\uprho }_{ {\mathrm{T}}_{{\mathrm{CH}}_{4}}}\cdot \frac{{\mathrm{V}}_{\mathrm{liq}}}{{\mathrm{V}}_{\mathrm{gas}}},$$

9 $$\frac{{\mathrm{dS}}_{\mathrm{gas},{\mathrm{CO}}_{2}}}{\mathrm{dt}}={\uprho }_{ {\mathrm{T}}_{{\mathrm{CO}}_{2}}}\cdot \frac{{\mathrm{V}}_{\mathrm{liq}}}{{\mathrm{V}}_{\mathrm{gas}}}.$$

Rates ${\rho }_{j}$ of the *j*th process and the stoichiometric coefficients ${\nu }_{i,j}$ of *i*th component in *j*th process of Eqs. (2)–(9) are explicated in the Petersen matrix below (Table 5).

**Table 5:** Petersen matrix of model components [29, 63, 64]

Process j	Component *i*								${\uprho }_{j},\mathrm{ rates}$ kg COD m^−3^ d^−1^
	${\mathbf{S}}_{{{\mathbf{ac}}}}$ kg COD m^−3^	${\mathbf{S}}_{{{\mathbf{CH}}4}}$ kg COD m^−3^	${\mathbf{S}}_{{{\mathbf{IC}}}}$ kmol C m^−3^	${\mathbf{X}}_{{{\mathbf{ac}}}}$ kg COD m^−3^	${\mathbf{S}}_{{{\mathbf{ac}} - }}$ kg COD m^−3^	${\mathbf{S}}_{{{\mathbf{HCO}}_{3} - }}$ kg COD m^−3^	${\mathbf{S}}_{{{\mathbf{gas}},{\mathbf{CH}}4}}$ kg COD m^−3^	${\mathbf{S}}_{{{\mathbf{gas}},{\mathbf{CO}}2}}$ kmol C m^−3^	
1 Uptake of acetate	− 1	$1 - Y_{ac}$	$- C_{ac} + \left( {1 - Y_{ac} } \right) C_{{CH_{4} }}$ $+ Y_{ac} C_{ac}$	$Y_{ac}$					${\text{k}}_{{{\text{m}},{\text{ac}}}} { }\left( {\frac{{{\text{S}}_{{{\text{ac}}}} }}{{{\text{K}}_{{{\text{S}},{\text{ac}}}} + {\text{S}}_{{{\text{ac}}}} }}} \right){\text{X}}_{{{\text{ac}}}}$
2 Decay of *X*_*ac*_				1					${\text{k}}_{{{\text{dec}},{\text{X}}_{{{\text{ac}}}} }} \cdot {\text{ X}}_{{{\text{ac}}}}$
${{A}}_{{{{ac}} - }}$ Acid–base acetate					− 1				${\text{k}}_{{{\text{A}}/{\text{Bac}}}} { }\left( {{\text{S}}_{{{\text{ac}} - }} \left( {{\text{S}}_{{{\text{H}} + { }}} + {\text{K}}_{{{\text{a}},{\text{ac}}}} } \right) - {\text{K}}_{{{\text{a}},{\text{ac}}}} \cdot {\text{S}}_{{{\text{ac}}}} } \right)$
${{A}}_{{{{HCO}}3 - }}$ Acid–base inorganic carbon						− 1			${\text{k}}_{{{\text{A}}/{\text{BCO}}_{2} }} { }({\text{S}}_{{{\text{HCO}}_{3}^{ - } }} \left( {{\text{K}}_{{{\text{a}},{\text{CO}}_{2} }} + {\text{S}}_{{{\text{H}}^{ + } { }}} } \right)$ $- {\text{ K}}_{{{\text{a}},{\text{CO}}_{2} }} \cdot {\text{S}}_{{{\text{IC}}}} )$
T_CH4_ Liquid–gas CH_4_ transfer		− 1					1		${\text{k}}_{{\text{L}}} {\text{a }} \cdot \left( {{\text{S}}_{{{\text{CH}}_{4} }} - 64{\text{ K}}_{{{\text{H}},{\text{CH}}_{4} { }}} \cdot {\text{p}}_{{{\text{CH}}4}} } \right)$^*^
T_CO2_ Liquid–gas CO_2_ transfer			− 1				1		${\text{k}}_{{\text{L}}} {\text{a }} \cdot \left( {{\text{S}}_{{{\text{IC}}}} - {\text{S}}_{{{\text{HCO}}_{3}^{ - } }} - {\text{K}}_{{{\text{H}},{\text{CO}}_{2} { }}} \cdot {\text{p}}_{{{\text{CO}}_{2} }} } \right)$

^*^The factor of 64 was used to convert the Henry’s law coefficient of CH_4_ ($K_{{{\text{H}},{\text{CH}}_{{4}} }}$) from mol L^−1^ bar^–1^ to kg COD m^–3^ bar^–1^, in order to account for the COD basis of *S*_CH4_ as compared to the molar basis of *K*_H_ [29]; $S_{{H^{ + } }}$ is the *H*^+^ concentration.

${p}_{{\mathrm{CH}}_{4}}$ and ${p}_{{\mathrm{CO}}_{2}}$ (bar) from Table 5 are CH_4_ and CO_2_ partial pressures in biogas, respectively, and are calculated according to the state equation of ideal gases reported in the following equations:

10 $${p}_{{\mathrm{CH}}_{4}}={S}_{\mathrm{gas},{\mathrm{CH}}_{4}}\cdot R\cdot {T}_{\mathrm{op}},$$

11 $${p}_{{\mathrm{CO}}_{2}}={S}_{\mathrm{gas}, {\mathrm{CO}}_{2}}\cdot R\cdot {T}_{\mathrm{op}}.$$

*R* is the ideal gas constant (bar L mol^−1^ K^−1^); ${T}_{\mathrm{op}}$ is the absolute temperature in the digester (K); ${V}_{\mathrm{liq}}$ and ${V}_{\mathrm{gas}}$ (m^3^) are the volume of the liquid and the volume of the gas in the reactor, respectively.

The concentration of hydrogen ions, ${S}_{{\mathrm{H}}^{+}}$, was determined through the charge balance.

All the parameters used in the model simulations and reported in Table 5 were assumed or calculated according to the scientific literature [29, 62–64], and their values are reported in Table 6.

**Table 6:** Parameters used in ADM1 simulations in the batch reactor [29, 62–64]

Parameter	Expression	Value	Unit
*C*_ac_	Carbon content of acetate	0.0313	kmol C (kg COD)^−1^
*C*_CH4_	Carbon content of methane	0.0156	kmol C (kg COD)^−1^
*K*_a,ac_	Acid dissociation constant of acetate	1.738∙10^–5^	mol L^−1^
*K*_a,CO2_	Acid dissociation constant of CO_2_ at temperature 303 K	4.700∙10^–7^	mol L^−1^
*k*_A/Bac_	Acid–base kinetic constant of acetate	1∙10^10^	L mol^−1^ d^−1^
*k*_A/Bco2_	Acid–base kinetic constant of CO_2_	1∙10^10^	L mol^−1^ d^−1^
*k*_dec*,*Xac_	First-order decay rate	0.0200–0.0400	d^−1^
*K*_H*,*CH4_	Henry’s law coefficient of CH_4_ at temperature 303 K	0.0013	mol L^−1^ bar^−1^
*K*_H,CO2_	Henry’s law coefficient of CO_2_ at temperature 303 K	0.0308	mol L^−1^ bar^−1^
*k*_*L*_*a*	Overall gas–liquid mass transfer coefficient	200*	d^−1^
*k*_*m,ac*_	Monod maximum specific uptake rate for acetate	4.100–7.800	kg COD kg COD ^−1^ d^−1^
*K*_S,ac_	Half-saturation value for acetate	0.0500–0.600	kg COD m^−3^
*K*_*w*_	Ion constant for water	1.450∙10^–14^	mol L^−1^
*R*	Ideal gas constant	0.0831	bar L mol^−1^ K^−1^
*T*_base_	Absolute temperature in standard condition	298.15	K
*T*_op_	Absolute temperature in digester	303.15	K
*Y*_ac_	Yield of biomass on acetate	0.0500	–

^*^Overall gas–liquid mass transfer coefficient for both CO_2_ and CH_4_

The initial amount of acetate substrate (*S*_ac_) was set equal to 3, 5 and 14 gCOD L^−1^, according to the amounts dosed in the experimental work performed by Lindeboom et al. [22]; the particulate composite matter (*X*_ac_) was assumed equal to 0.5% of the total sodium acetate substrate; ${S}_{\mathrm{gas},{\mathrm{CH}}_{4}}$ and ${S}_{\mathrm{gas},{\mathrm{CO}}_{2}}$ were zero. Simulations were performed by implementing the model equation system in the MATLAB environment (MATLAB R2019b).

### Model calibration and validation

ADM1 was previously implemented in many software packages WEST, GPS-X, SIMBA and Aquasim [65]. In this work, the ordinary differential equations of ADM1 were coded and implemented using MATLAB/Simulink and integrated with the ODE15s solvers, which solve stiff ODE systems. The MATLAB/Simulink was found to be adaptable for further structural modifications to enable integration of the model with other blocks containing other elements and to monitor the evolution of each variable in the course of a simulation in real-time throughout an output block [56].

Kinetic parameters are of great significance in determining the predictability of the model and, consequently, for the proper design of the PAD reactor. In particular, a mathematical calibration was performed through a Genetic Algorithm (GA) using MATLAB R2019b Global Optimization Toolbox, which is an effective method for the modelling with ADM1, to further improve the fitting of kinetic parameters and to identify their ranges, since GA includes concepts of biological evolution processes, like heredity, selection and mutation [32]. GA allowed minimising a fitness value function of the sum of differences between the experimental data and the simulation results. The fitness function considered is expressed below:

12 $$\mathrm{Fitness function}=\sqrt{\sum {\frac{({P}_{\mathrm{exp} }-{ P}_{\mathrm{sim}})}{N}}^{2},}$$

where *P*_exp_, *P*_sim_ and *N* are the experimental pressure results, the simulated pressure results, and the total number of experiments, respectively.

Minimising the objective function is an important issue for prediction purposes or process stability. Model calibration was carried out for assessing *k*_m,ac_, *K*_S,ac_ and *k*_dec,*X*ac_.

Few experimental results about the effects of time and pressure on PAD performance are reported in the literature. A very comprehensive study was presented by Lindeboom et al. [22], in which the effects of these parameters on an AHPD process, using sodium acetate as substrate, were investigated. All experiments were carried out in a lab-scale batch autogenerative high-pressure reactor without agitation, at the temperature of 303.15 K, by varying the initial concentration of sodium acetate and the headspace volume of the reactor, i.e. the gas volume in the reactor. An increase in autogenerative pressure was investigated in the safe range of operation for the reactors 0–90 bar. Operative conditions used for the calibration and validation of the proposed model are reported in Table 7. Experiments were carried out using disintegrated granular sludge as inoculum, collected from the UASB plant of Eerbeek (The Netherlands) papermill factory, and sodium acetate as mono-substrate [22].

**Table 7:** Overview of Lindeboom et al. [22] experiments considered for the present work

Experiment No.	Reactor volume (L)	Gas volume (L)	Substrate (g sodium acetate COD/L)	Run time (h)
4	1.68	0.04	3	160
5	1.68	0.04	5	60
6	1.68	0.01	14	96
7	1.68	0.01	14	170

During the experimental investigations, the pH was monitored, and a constant value of about 7 was found. The absence of variation of the pH can be explained by considering a constant ratio between acid neutralising capacity (ANC) and total inorganic carbon produced (ANC/TIC) as was experimentally highlighted by Lindeboom et al. [66].

Model calibration was carried out by using the experimental pressure increase as a function of time until the experimental run time of 160 h was reached, conforming to Experiment No. 7 performed by Lindeboom et al. [22], with the initial pH of 7. The parameters *k*_m,ac_, *K*_S,ac_ and *k*_dec,*X*ac_ were varied until the fitness function (Eq. 12) was minimised.

Results from experiments No. 4, 5, 6, and 7, in terms of final pressure, CH_4_ biogas molar fraction and SMY, reported in Lindeboom et al. [22], were used to validate the model. The SMY was calculated according to the following equation [67]:

13 $$SMY=\frac{{\mathrm{n}}_{\mathrm{gas},\mathrm{CH}4}\mathrm\,{ R }{\mathrm{T}}_{\mathrm{STP}}}{{\mathrm{P}}_{\mathrm{STP}} \,{\mathrm{S}}_{\mathrm{COD added}}},$$

where ${n}_{\mathrm{gas},{\mathrm{CH}}_{4}}$ is the CH_4_ mole number in accumulated gas (kmol), *T*_STP_ is the standard temperature (273.15 K) and *P*_STP_ standard pressure (1 bar), *R* is the ideal gas constant (0.083145 bar M^−1^ K^−1^), and ${S}_{\mathrm{COD added}}$ (kg COD) is the mass of COD added in the reactor.

It is worth highlighting that the proposed approach may allow to potentially identify the aceticlastic methanogens species able to grow at high pressures. For example, the value of the half-saturation constant for acetate depends on which aceticlastic methanogens would predominate, i.e. *Methanosarcina* vs *Methanosaeta*, that respond with different sensitivity to the environmental stress, with the typical values (order of magnitude) of 0.03 kg COD/m^3^ and 0.3 kg COD/m^3^, respectively [40, 43].

### Sensitivity analysis

A parametric sensitivity analysis was performed to define the most sensitive ADM1 parameters during the production of biogas and consumption of acetate.

Sensitivity analysis was carried out by varying one parameter in a specific range, while the other parameters were kept constant at the values assessed with the model calibration process. For this aim, simulations were carried out by varying *k*_m,ac,_ *K*_S,ac_, *k*_dec*,X*ac,_ in typical ranges retrieved in the scientific literature related to AD at atmospheric pressure [68]. In particular, *k*_m,ac_ was varied in the range 4.1–7.8 kg COD·kg COD^−1^·d^−1^; *K*_S,ac_ in the range 0.05–0.6 kg COD m^−3^; *k*_dec*,X*ac_ in the range 0.02–0.04 d^−1^ [68]. Also, the mass transfer coefficient, ${k}_{L}a$, was varied in the range 1–1000 d^−1^, to assess its effect on model performance and results. Detailed simulation plans of the sensitivity analysis are shown in Tables 8 and 9.

**Table 8:** Simulation plan of the sensitivity analysis: *k*_m,ac_, *K*_S,ac_, *k*_dec*,X*ac_

Scenario	*k*_*m,ac*_ (kg COD·kg COD ^−1^ d^−1^)	*K*_*S,ac*_ (kg COD m^−3^)	*k*_*dec,Xac*_ (d^−1^)	*k*_*L*_*a* (d^−1^)
A	4.1	0.05	0.02	200
B	5.3	0.05	0.02	200
C	5.9	0.05	0.02	200
D	6.5	0.05	0.02	200
E	7.8	0.05	0.02	200
F	5.9	0.055	0.02	200
G	5.9	0.1	0.02	200
H	5.9	0.3	0.02	200
I	5.9	0.6	0.02	200
L	5.9	0.05	0.022	200
M	5.9	0.05	0.03	200
N	5.9	0.05	0.04	200

**Table 9:** Simulation plan of the sensitivity analysis: *k*_L_*a*

Scenario	*k*_*m,ac*_ (kg COD·kg COD ^−1^ d^−1^)	*K*_*S,ac*_ (kg COD m^−3^)	*k*_*dec,Xac*_ (d^−1^)	*k*_*L*_*a* (d^−1^)
O	5.9	0.05	0.02	1
P	5.9	0.05	0.02	200
Q	5.9	0.05	0.02	1000

## Data Availability

All data generated or analysed during this study are included in this published article.

## Abbreviations

AHPD: Autogenerative high-pressure anaerobic digestion
ADM1: Anaerobic digestion model No.1
AD: Anaerobic digestion
PAD: Pressurised anaerobic digestion
SMY: Specific methanogenic yield
AA: Amino acids
LCFA: Long-chain fatty acids
MS: Monosaccharides
ANC: Acid neutralising capacity
TIC: Total inorganic carbon
GA: Genetic algorithm
